# Acute Hypotensive Effects of 2-Acetylfuran and 5-Methylfurfural and Their Impact on Liver Mitochondrial Bioenergetics

**DOI:** 10.3390/ph19070995

**Published:** 2026-06-26

**Authors:** Irma Martišienė, Jurgita Šapauskienė, Dominyka Adamonė, Ieva Lankutytė, Rasa Banienė, Vilma Zigmantaitė, Jonas Jurevičius, Regina Mačianskienė

**Affiliations:** 1 Laboratory of Membrane Biophysics, Institute of Cardiology, Lithuanian University of Health Sciences, Sukilėlių Ave. 15, LT50162 Kaunas, Lithuania; dominyka.adamone@lsmu.lt (D.A.); vilma.zigmantaite@lsmu.lt (V.Z.); jonas.jurevicius@lsmu.lt (J.J.); regina.macianskiene@lsmu.lt (R.M.); 2 Department of Biochemistry, Lithuanian University of Health Sciences, Eivenių St. 4, LT50161 Kaunas, Lithuania; jurgita.sapauskiene@lsmu.lt (J.Š.); rasa.baniene@lsmu.lt (R.B.); 3 Biological Research Center, Lithuanian University of Health Sciences, Tilžės St. 18/7, LT47181 Kaunas, Lithuania

**Keywords:** furan compounds, hypotensive effect, mitochondrial inhibition, 2-acetylfuran, 5-methylfurfural

## Abstract

**Background/Objectives**: Furan derivatives are commonly encountered in food and environmental matrices and may exert biological effects, but their acute cardiovascular actions and potential mitochondrial targets remain insufficiently characterised. This study examined the effects of two simple furan compounds, 2-acetylfuran (2AF) and 5-methylfurfural (5MFF), on arterial blood pressure in vivo and on oxidative phosphorylation in isolated rat liver mitochondria. **Methods**: Arterial blood pressure was recorded invasively in anaesthetised rats after intraperitoneal administration of 2AF or 5MFF (0.3 µL/g). Systolic, diastolic, and mean arterial pressures, as well as heart rate, were monitored over time. Mitochondrial respiration was assessed in isolated rat liver mitochondria using high-resolution respirometry. **Results**: Both 2AF and 5MFF induced a rapid hypotensive response, with significant reductions in systolic, diastolic, and mean arterial pressures within 10–15 min after administration. MAP was reduced to a similar extent by both compounds. However, their chronotropic and pulse pressure responses differed: 5MFF increased heart rate and pulse pressure, whereas 2AF induced a delayed bradycardic response without a significant change in pulse pressure. In isolated liver mitochondria, both compounds markedly reduced ADP-stimulated respiration and decreased the respiratory control index, indicating reduced coupling efficiency. Both compounds also increased the cytochrome c effect, suggesting partial impairment of outer mitochondrial membrane integrity. **Conclusions**: 2AF and 5MFF exert acute hypotensive effects in anaesthetised rats and impair oxidative phosphorylation in isolated rat liver mitochondria. This study provides the first in vivo evidence that 2AF and 5MFF exert hypotensive effects and identifies them as bioactive furan compounds with dual haemodynamic and bioenergetic actions.

## 1. Introduction

Cardiovascular diseases remain a major cause of morbidity and mortality worldwide. Arterial hypertension, characterised by persistently elevated blood pressure, contributes to structural and functional injury of the heart, kidney, and vasculature and is a major risk factor for stroke, coronary artery disease, and heart failure [1,2]. Although substantial progress has been made in antihypertensive therapy, blood pressure regulation remains an important target in cardiovascular research. In this context, the identification of bioactive compounds that modulate vascular tone or haemodynamic function is relevant both to understanding cardiovascular physiology and to assessing the biological impact of diet- and environment-associated molecules.

Natural compounds are an important source of cardiovascular modulators. For example, the essential oil of *Elsholtzia ciliata* has been shown to exert antiarrhythmic effects [3] and to lower arterial blood pressure in vivo [4]. Phytochemical analysis revealed that its main constituents are ketones containing a furan ring [5]. These findings suggest that furan-containing compounds may contribute to the cardiovascular actions observed in plant-derived preparations.

The furan ring is a heterocyclic structure widely distributed in natural and synthetic molecules. Furan derivatives are used in pharmacology due to their diverse activities, including anti-inflammatory, antifungal, antihyperglycemic, and antineoplastic effects [6]. They are also abundant as flavor compounds in food, formed during the thermal processing of carbohydrates and amino acids [7,8,9,10]. At the same time, the toxicological properties of furan derivatives, such as hepatotoxicity and genotoxicity, have been extensively described, particularly for 5-Hydroxymethylfurfural and furfural [11,12]. Thus, furan derivatives are of interest not only as bioactive food- and plant-associated compounds but also as molecules with potential systemic toxicodynamic effects.

Recent in vitro studies have shown that 2-acetylfuran (2-Furyl methyl ketone, 2AF) and 5-methylfurfural (5-Methylfuran-2-carbaldehyde, 5MFF) exert a smooth muscle relaxant effect in rat aorta and prostate preparations [13]. Since vascular smooth muscle tone is a critical determinant of arterial blood pressure, these findings suggest that 2AF and 5MFF may exert hypotensive effects in vivo. However, direct evidence linking these in vitro vascular effects to acute cardiovascular effects in intact animals is lacking.

Mitochondria play a crucial role in energy metabolism and serve as important regulators of cardiovascular physiology. They provide ATP for cellular contraction, regulate reactive oxygen species, and govern cell survival pathways [14,15]. The liver, as the major site of xenobiotic metabolism, is particularly relevant for evaluating how small molecules affect mitochondrial respiration. Assessing the mitochondrial actions of 2AF and 5MFF therefore provides complementary insight into their biological activity.

The present study aimed to determine whether 2AF and 5MFF induce acute changes in arterial blood pressure in rats and to evaluate their effects on oxidative phosphorylation in isolated rat liver mitochondria. By combining invasive hemodynamic monitoring with high-resolution mitochondrial respirometry, this study provides new evidence on the in vivo cardiovascular actions of selected furan derivatives and their associated bioenergetic effects.

## 2. Results

### 2.1. Cardiovascular Effects of 5MFF and 2AF In Vivo

The mean arterial pressure (MAP) of anaesthetised rats before injection was 96.6 ± 11.0 mmHg (*n* = 5) in the 5MFF group and 105.6 ± 10.5 mmHg (*n* = 4) in the 2AF group (Table 1). After intraperitoneal administration (0.3 µL/g), both compounds induced a rapid and marked decrease in MAP that began within 30 s of injection. 5MFF induced a gradual decline in MAP, reaching a minimum value at 15 min with a 37% reduction compared with the pre-injection value (*p* < 0.05). Thereafter, MAP remained at a similarly reduced level throughout the 30-min observation period. Under administration of 2AF, MAP fell more sharply, reaching its lowest value at 10 min (43% decrease vs. pre-injection value), but then showed a gradual upward trend. Overall, both 2AF and 5MFF significantly reduced MAP, with the greatest hypotensive effect observed within 10–15 min after administration. MAP was reduced to a similar extent by both compounds. Representative haemodynamic recordings obtained under control conditions and following 5MFF administration are provided in Supplementary Figure S1. Under control conditions, only minor time-dependent fluctuations in haemodynamic parameters were observed, whereas administration of 5MFF was followed by a characteristic decrease in arterial pressure and an increase in heart rate.

**Figure 1:**
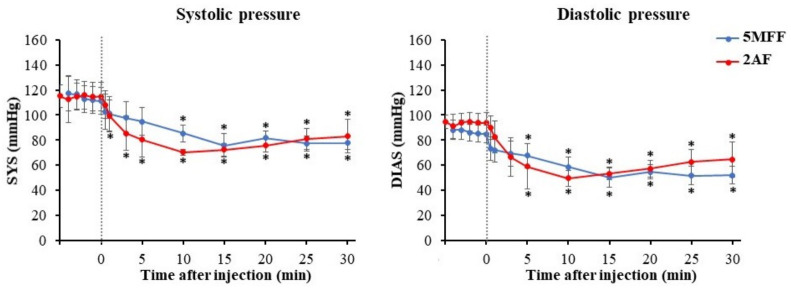
Effects of 5-methylfurfural (5MFF) and 2-acetylfuran (2AF) on systolic and diastolic arterial pressure in anaesthetised rats. The negative time points represent the pre-injection baseline period, and the dashed vertical line indicates the time of compound administration. Data are presented as mean ± SEM (*n* = 4–5 per group), * *p* < 0.05 vs. pre-injection values.

The systolic (SYS) and diastolic (DIAS) arterial pressure profiles (Figure 1) closely followed the changes observed in MAP. The SYS and DIAS of anaesthetised rats before injection were respectively 111.3 ± 10.5 mmHg and 86.2 ± 6.8 mmHg (*n* = 5) in the 5MFF group and 114.6 ± 11.3 mmHg and 93.9 ± 7.7 mmHg (*n* = 4) in the 2AF group. After administration of either 5MFF or 2AF, both systolic and diastolic pressures decreased rapidly and to a similar extent.

**Figure 2:**
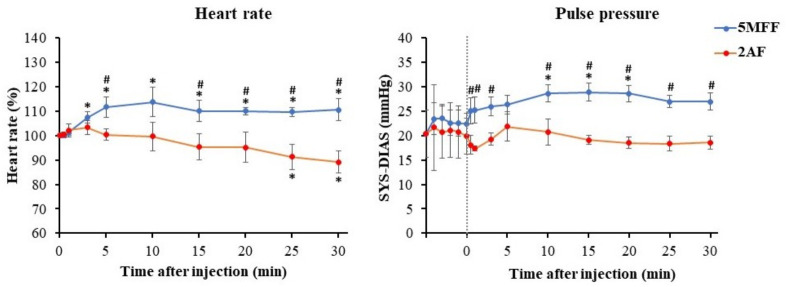
Effects of 2-acetylfuran (2AF) and 5-methylfurfural (5MFF) on heart rate and pulse pressure (SYS–DIAS) in anaesthetised rats. The negative time points represent the pre-injection baseline period, and the dashed vertical line indicates the time of compound administration Data are expressed as mean ± SEM (*n* = 4–5 per group), * *p* < 0.05 vs. baseline; # *p* < 0.05 vs. corresponding 2AF value.

The effects of 2AF and 5MFF on heart rate and pulse pressure (SYS–DIAS) are presented in Figure 2. The heart rate of anesthetized rats was 205 ± 21 beats per min (ranging from 170 to 236 beats per min). The effects of the tested compounds on heart rate were evaluated by percentage changes relative to baseline. Following intraperitoneal administration of 5MFF, heart rate increased significantly within the first 5–10 min by approximately 10–13% above baseline (*p* < 0.05) and remained elevated throughout the 30-min recording period. In contrast, heart rate was not significantly affected during the first 10 min after 2AF administration, but subsequently decreased gradually, reaching about 10–12% below baseline at 30 min (*p* < 0.05 vs. baseline). Thus, although both compounds lowered arterial pressure, 5MFF induced a reflex tachycardia, whereas 2AF caused a delayed bradycardic response.

Pulse pressure, calculated as the difference between systolic and diastolic pressure, was evaluated as an indirect haemodynamic index reflecting changes in the relationship between systolic and diastolic pressure. The pulse pressure of anaesthetised rats before injection was 22.5 ± 2.2 mmHg (*n* = 5) in the 5MFF group and 20.7 ± 5.4 mmHg (*n* = 4) in the 2AF group. After 5MFF administration, pulse pressure increased to 28.9 ± 1.9 mmHg at 15 min and remained elevated to 27 ± 1.7 mmHg at 30 min (*p* < 0.05 vs. pre-injection), indicating a relatively greater reduction in diastolic than in systolic pressure. After 2AF administration, there was no significant change in pulse pressure.

### 2.2. Effects of 5MFF and 2AF on Oxidative Phosphorylation of Liver Mitochondria

Because the liver is a major site of xenobiotic metabolism and mitochondrial function is essential for cellular energy homeostasis, the next stage of the study examined the effects of 2AF and 5MFF on oxidative phosphorylation of isolated rat liver mitochondria.

**Figure 3:**
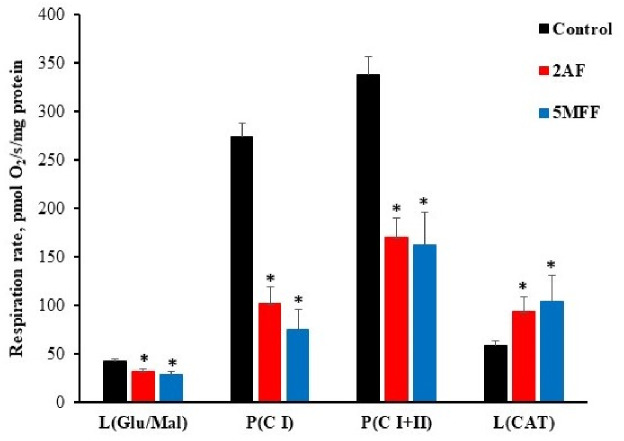
Effects of 2-acetylfuran (2AF) and 5-methylfurfural (5MFF) on the respiration rate of isolated rat liver mitochondria in different metabolic states. L(Glu/Mal)—leak respiration after addition of glutamate and malate; P(C I)—ADP-stimulated oxidative phosphorylation through complex I; P(C I+II)—maximal oxidative phosphorylation after addition of both complex I and II substrates, glutamate/malate and succinate; L(CAT)—non-phosphorylating respiration after addition of carboxyatractyloside. Values are mean ± SEM (*n* = 5–7 per group), * *p* < 0.05 vs. control.

The effects of 2AF and 5MFF on mitochondrial respiration in different metabolic states are presented in Figure 3. In control mitochondria, the respiration rate during oxidation of glutamate and malate in the leak state L(Glu/Mal) was 41.8 ± 2.9 pmol O_2_/s/mg protein. In the presence of 2AF and 5MFF, this rate was significantly decreased by 24% and 32%, respectively (*p* < 0.05 vs. control). During ADP-stimulated oxidative phosphorylation through mitochondrial respiratory chain complex I (P(C I)), respiration in control mitochondria reached 273.2 ± 14.1 pmol O_2_/s/mg protein; under exposure of 2AF this rate was lower by 63%, and 5MFF by 73% vs. control (*p* < 0.05). When both complex I and complex II substrates were supplied (P(C I+II)), the respiration rate was 338.1 ± 18.4 pmol O_2_/s/mg protein in the control, but was lower by 50% after 2AF and by 52% after 5MFF treatment (*p* < 0.05). After the addition of carboxyatractyloside, respiration in the L(CAT) state was 58.7 ± 4.4 pmol O_2_/s/mg protein in control mitochondria. In the presence of 2AF and 5MFF, L(CAT) respiration was significantly increased by 58% and 77%, respectively, compared with control mitochondria (*p* < 0.05).

**Figure 4:**
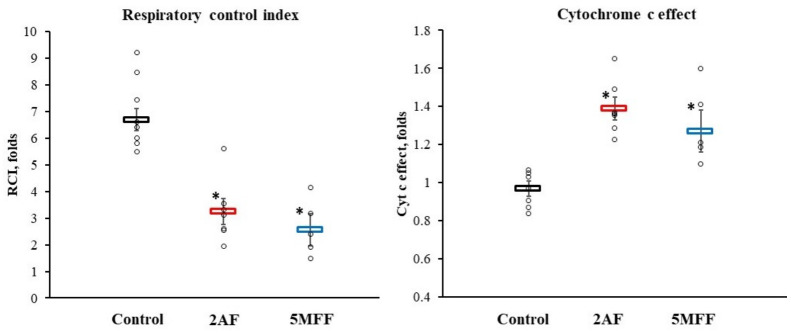
Effects of 2-acetylfuran (2AF, red bars) and 5-methylfurfural (5MFF, blue bars) on the respiratory control index (RCI) and cytochrome c (Cyt c) effect in rat liver mitochondria. Open circles represent individual observations, and horizontal bars indicate mean ± SEM. * *p* < 0.05 vs. control.

The respiratory control index (RCI) and cytochrome c effect are presented in Figure 4. In control mitochondria, RCI was 6.7 ± 0.4, whereas in mitochondria exposed to 2AF or 5MFF it decreased to 3.3 ± 0.5 and 2.6 ± 0.6, respectively (*p* < 0.05 vs. control). The cytochrome c effect, which reflects the permeability of the outer mitochondrial membrane, was significantly increased after exposure to either compound. In control mitochondria, the cytochrome c effect was 0.97 ± 0.04, while after treatment with 2AF and 5MFF it increased to 1.39 ± 0.06 and 1.27 ± 0.11, respectively (*p* < 0.05 vs. control).

## 3. Discussion

The present study demonstrates that the furan derivatives 2-acetylfuran (2AF) and 5-methylfurfural (5MFF) induce a rapid and significant reduction in arterial blood pressure in vivo and markedly alter oxidative phosphorylation in isolated rat liver mitochondria. These findings provide the first in vivo evidence that these simple furan derivatives exert acute hypotensive effects. In addition, the mitochondrial data show that both compounds have direct bioenergetic activity, which may be relevant to their broader systemic toxicodynamic profile.

Both 2AF and 5MFF produced an immediate and pronounced decrease in arterial blood pressure, with maximum effects observed within 10–15 min of administration. The magnitude of the response was comparable between compounds. The speed of onset of vascular effect is likely to involve smooth muscle relaxation rather than slower neurohumoral pathways. Similar hypotensive profiles have been demonstrated for *E. ciliata* essential oil, which contains furan-ring ketones and has been shown to reduce smooth muscle contractility and arterial pressure in animal models [4]. Various furan derivatives have been reported to induce vascular relaxation by inhibiting calcium influx, activating potassium channels, or modulating cyclic nucleotide signalling pathways. Activation of calcium-activated or ATP-sensitive potassium channels, such as BK_Ca_ and K_ATP_, causes membrane hyperpolarisation, reduces the opening of L-type Ca^2+^ channels, and lowers intracellular calcium concentration, leading to vasodilation [16,17,18]. Furan-2-carbaldehyde, also known as furfural, has been shown at high concentration to induce vasodilation in isolated rat mesenteric resistance arteries by enhancing K^+^ efflux and inhibiting calcium entry [19]. Similarly, 5-hydroxymethylfurfural has been reported to relax coronary arteries and inhibit L-type Ca^2+^ channels in cardiac myocytes [20]. Tetrahydrofuran-ring lignans, such as sesamin, have also been shown to enhance endothelial nitric oxide synthesis and increase cGMP levels in vascular endothelium models [21]. In line with these observations, our previous study demonstrated that both 2AF and 5MFF reduced phenylephrine-induced contractions in rat aorta and prostate smooth muscle, suggesting interference with α_1_-adrenergic-mediated calcium mobilization [13]. Taken together, these findings support the hypothesis that the hypotensive effects of 2AF and 5MFF may involve modulation of vascular smooth muscle tone. However, the precise molecular targets remain to be identified.

Both compounds lowered arterial pressure; their effects on heart rate diverged. 5MFF increased heart rate, which is consistent with reflex tachycardia due to baroreceptor activation following a fall in blood pressure [22]. This pattern suggests that 5MFF acts primarily via peripheral vasodilation without directly inhibiting cardiac automaticity or sympathetic drive. 2AF decreased heart rate, producing mild bradycardia. This may reflect direct depression of sinoatrial node excitability, inhibition of cardiac ion channels, or enhancement of vagal activity. *E. ciliata* oil, containing structurally related furan ketones, has been shown to inhibit cardiac sodium channels and prolong conduction [3], supporting the hypothesis that 2AF may act via similar electrophysiological mechanisms. These different chronotropic responses highlight that small structural differences between simple furan derivatives can profoundly alter their autonomic and cardiac profiles. The pulse pressure data further support this interpretation. After 5MFF administration, pulse pressure increased, indicating a relatively greater reduction in diastolic than systolic pressure. This pattern is compatible with a stronger effect on peripheral vascular tone or other determinants of diastolic pressure. In contrast, 2AF did not significantly change pulse pressure, despite reducing MAP. These findings suggest that, although the overall hypotensive effect of both compounds was comparable, the relative contribution of vascular and cardiac components may differ.

The different haemodynamic profiles of 2AF and 5MFF may also be related to their distinct carbonyl chemistry. Both compounds contain a furan ring, but they differ in the nature of the carbonyl group: 2AF is a furan ketone, whereas 5MFF is a furan aldehyde. This structural distinction may influence their chemical reactivity, metabolism, and interactions with cellular targets. Aldehydes are generally more electrophilic than ketones and may interact more readily with nucleophilic cellular targets, including proteins and redox-sensitive signalling pathways. Therefore, the aldehyde group of 5MFF may contribute to its distinct haemodynamic profile. However, the present study did not directly assess compound metabolism, protein adduct formation, oxidative stress, or specific vascular and cardiac molecular targets. Thus, the role of the aldehyde group in 5MFF and the ketone group in 2AF remains a mechanistic hypothesis requiring further investigation.

In this study, arterial blood pressure was recorded in anaesthetised rats. This approach provides stable conditions for invasive haemodynamic measurements and reduces stress-related variability. However, ketamine–xylazine anaesthesia may reduce sympathetic tone, heart rate, and baseline blood pressure compared with the conscious state [23,24,25]. Baroreflex-mediated cardiovascular adjustments may still be present under ketamine-based anaesthesia, but autonomic reflexes and vascular responses can be attenuated or modified [26]. Therefore, the magnitude and pattern of haemodynamic responses observed in this study may differ from those in conscious animals. Consequently, the divergent chronotropic responses observed after 5MFF and 2AF administration should be interpreted with caution, as ketamine–xylazine anaesthesia may have influenced autonomic and baroreflex-mediated regulation of heart rate. Future studies using telemetry in freely moving rats, as well as experiments in hypertensive models and with different dose levels, would be valuable to determine whether the hypotensive effects of 2AF and 5MFF are preserved under conditions of elevated vascular tone and to further characterize their cardiovascular pharmacological profile.

The liver mitochondrial experiments were performed to assess the bioenergetic effects of 2AF and 5MFF, because the liver is a major site of xenobiotic metabolism and is particularly vulnerable to mitochondrial toxicants [27]. However, mitochondrial function exhibits marked tissue-specific heterogeneity. Mitochondria from different organs differ in oxidative phosphorylation capacity, substrate utilisation, calcium handling, and susceptibility to toxic injury. In particular, cardiac mitochondria possess substantially higher oxidative capacity and ATP turnover rates than liver mitochondria because of the continuous energetic demands of myocardial contraction [28]. Therefore, the effects observed in isolated liver mitochondria cannot be directly extrapolated to cardiovascular tissues. Nevertheless, liver mitochondria represent a relevant model for the initial assessment of mitochondrial liabilities of xenobiotics because the liver is the primary organ responsible for biotransformation and systemic exposure to reactive metabolites.

Both compounds markedly reduced ADP-stimulated respiration, and this inhibition persisted when both complex I- and complex II-linked substrates were supplied. This pattern suggests that inhibition of complex I alone may not fully explain the observed reduction in respiration. However, because TMPD/ascorbate-supported respiration was not assessed, the possible involvement of cytochrome c oxidase could not be evaluated. Therefore, the precise site of inhibition within the oxidative phosphorylation system remains unresolved. In parallel, both compounds significantly reduced the respiratory control index, showing that the efficiency of coupling between substrate oxidation and ADP phosphorylation was decreased [29]. The increase in respiration after the addition of carboxyatractyloside (L(CAT)) further supports this interpretation. Because carboxyatractyloside inhibits adenine nucleotide translocase and blocks ADP/ATP exchange, the elevated L(CAT) observed under exposure to 2AF and 5MFF reflects increased non-phosphorylating oxygen consumption rather than improved mitochondrial function. In addition, both compounds increased the cytochrome c effect, suggesting partial impairment of outer mitochondrial membrane integrity. The mechanism underlying the apparent increase in outer mitochondrial membrane permeability remains unclear. Potential explanations include direct interactions with mitochondrial membrane components, alterations in membrane lipid organization, or secondary effects associated with impaired oxidative phosphorylation and mitochondrial dysfunction [30]. Taken together, our findings indicate that 2AF and 5MFF impair mitochondrial oxidative phosphorylation and affect mitochondrial membrane-associated functions in isolated liver mitochondria. Such alterations are consistent with mitochondrial dysfunction and may indicate a potential for impaired cellular bioenergetics and hepatocellular toxicity upon systemic exposure. Although the hepatic mitochondrial dysfunction observed in vitro does not directly account for the rapid hypotensive effect seen in vivo, it may reflect a general capacity of these compounds to interfere with mitochondrial function in other tissues, including the cardiovascular system. The onset of hypotension within seconds of administration indicates that the immediate haemodynamic response was not due to hepatic ATP depletion. Nevertheless, if similar mitochondrial inhibition occurs in vascular smooth muscle or endothelial cells, it could modulate vascular tone through secondary mechanisms such as altered redox signaling, decreased ATP-dependent calcium handling, or activation of potassium channels that promote membrane hyperpolarisation and vasorelaxation [16,31,32,33]. Under such circumstances, moderate impairment of oxidative phosphorylation can paradoxically promote vasodilatory signalling rather than vasoconstriction. Thus, while the liver mitochondrial data do not explain the immediate hypotensive response, they support the possibility that mitochondrial perturbation may contribute to the overall pharmacodynamic profile of these furan derivatives.

From a toxicological perspective, the observed inhibition of mitochondrial respiration raises concerns about the potential hepatotoxicity of 2AF and 5MFF, consistent with previous reports of furan and substituted furfural derivatives inducing mitochondrial injury and concomitant decrease in cellular ATP [34,35]. Furan and its substituted analogues can undergo metabolic activation via cytochrome P450 enzymes to form reactive intermediates, such as cis-2-butene-1,4-dial, capable of covalently modifying proteins and mitochondrial components [12,36,37,38]. While transient mitochondrial inhibition might contribute to pharmacological vasorelaxation, sustained or repeated exposure could lead to oxidative stress, energy failure, and hepatotoxicity. Further studies are warranted to determine whether the oxidative phosphorylation impairment observed in liver mitochondria is reversible and to elucidate the specific mitochondrial targets involved. Collectively, the current findings highlight mitochondrial toxicity as an important mechanistic component of the biological activity of 2AF and 5MFF, warranting careful consideration in the safety evaluation of these furan compounds.

Because 2AF and 5MFF are flavour- and fragrance-related furan compounds that may occur in food and smoke-related matrices, their toxicological relevance may extend beyond the experimental setting. Repeated low-level exposure to such compounds may be biologically relevant, particularly in sensitive individuals or under conditions of increased susceptibility. At the same time, the rapid haemodynamic effects observed in this study suggest that simple furan derivatives possess structural features capable of influencing vascular tone; however, their toxicological properties must be fully characterised before any pharmacological relevance or application can be considered.

## 4. Materials and Methods

### 4.1. Animals

The study was carried out in accordance with the EU Directive 2010/63/EU [39] on the protection of animals used for scientific purposes. Adult male Wistar rats (300–400 g) were used. The animals were housed in a controlled environment with appropriate conditions, i.e., humidity, temperature, light–dark cycle, and free access to food and water. The study was approved by the Lithuanian Commission on the Ethics of the Use of Experimental Animals at the State Food and Veterinary Service under Permission No. G2-199.

### 4.2. Materials and Chemicals

5-methylfurfural (99%), 2-acetylfuran (99%), and all other chemicals were purchased from Sigma-Aldrich (Schnelldorf, Germany).

### 4.3. Invasive Arterial Blood Pressure Measurement

The rats were not fasted before anaesthesia. Anaesthesia was induced with ketamine (10 mg/kg IP) and xylazine (90 mg/kg IP), and buprenorphine (0.05 mg/kg IP) was administered for analgesia. Once the animal relaxed, reflexes were assessed; when the animal no longer responded to painful or environmental stimuli, the fur on the ventral side of the neck—from the chin to the sternum and to the medial aspects of the forelimbs—was shaved. The fur on the medial aspects of the thighs was also shaved. The animal was then placed on a surgical table prepared for rodents. The table surface was electrically non-conductive to reduce environmental noise during arterial blood pressure recording.

A longitudinal skin incision was made along the ventral neck, and the neck muscles were bluntly separated until the carotid artery (*a. carotis communis*) was exposed, with the vagus nerve (*n. vagus*) visualised alongside it. The artery was carefully isolated with minimal contact with the vagus nerve. A ligature was placed cranially on the artery near the bifurcation, and another ligature was positioned caudally but left untied. The artery was gently lifted, and a 26G catheter (DISPOFLON, Disposafe Health and Life Care Ltd., Faridabad, Haryana, India) was inserted. The caudal ligature was then secured around the artery and catheter cannula to prevent bleeding. The catheter was also fixed cranially to keep it in place. A 22G connector was attached to a flexible tube (PE/PVC Extension line, Cair L.G.L., Lissieu, France) filled with heparinised saline (0.5 IU/mL), which was connected to a membrane transducer (Memscap 844-28 Dome, Memscap AS, Skoppum, Norway) [40,41].

After catheterisation, a stabilisation period of approximately 10 min was allowed until the recorded parameters became steady. Then, 2AF or 5MFF were administered intraperitoneally at a volume of 0.3 µL/g body weight, corresponding to 329 mg/kg (2.99 mmol/kg) and 332 mg/kg (3.02 mmol/kg), respectively. Changes in blood pressure and heart rate were recorded over 30 min. The selected dose was based on our previous study demonstrating relaxant effects of both compounds on rat smooth muscle and inhibition of phenylephrine-induced contractions [13]. At the end of the experiment, the rats were killed.

Arterial blood pressure parameters were analysed using LabChart Pro v8.1.16 with the Blood Pressure Module (ADInstruments, Colorado Springs, CO, USA).

### 4.4. Isolation of Liver Mitochondria

Rats were euthanised by gradually increasing the concentration of CO_2_, followed by cervical dislocation. The liver was rapidly excised and immersed in a cooled (0–4 °C) 0.9% KCl solution. Mitochondria were isolated using differential centrifugation. The tissue was minced with scissors and homogenised using a T10 basic ULTRA-TURRAX disperser (IKA, Stau-fen, Germany) in an isolation medium containing 250 mM sucrose, 10 mM Tris-HCl, 2 mM EGTA, and 1 mg/mL bovine serum albumin (BSA) (pH 7.7, 2 °C). The liver homogenate was centrifuged at 750× *g* for 5 min at 4 °C. The resulting supernatant was filtered through double-layered gauze and centrifuged at 10,000× *g* for 10 min at 4 °C. The supernatant was discarded, and the mitochondrial pellet was resuspended in a medium containing 250 mM sucrose and 5 mM Tris-HCl (pH 7.3, 2 °C). The isolated mitochondrial suspension was maintained on ice until further analysis. The protein concentration in the liver mitochondrial suspension was determined by the biuret method using bovine serum albumin as a standard [42].

### 4.5. Registration of Mitochondrial Respiration Rate

Mitochondrial respiration (oxygen consumption) was assessed using a high-resolution respirometry system (Oxygraph-2k, OROBOROS Instruments, Innsbruck, Austria) at 37 °C. Measurements were performed in a medium containing 0.5 mM EGTA, 3 mM MgCl_2_, 60 mM potassium lactobionate, 20 mM taurine, 10 mM KH_2_PO_4_, 20 mM HEPES, and 110 mM sucrose (pH 7.1 at 37 °C). Oxygen consumption rates were normalized to mitochondrial protein content and expressed as pmol O_2_/s/mg protein.

Freshly isolated rat liver mitochondria were added to the respirometry chamber, and the effects of 2AF and 5MFF (0.3 µL/mL) were assessed by direct addition of the compounds during the experimental protocol. Control mitochondria were analysed under identical experimental conditions without exposure to the tested compounds.

Leak respiration supported by complex I substrates was initiated by the addition of glutamate (5 mM) and malate (2 mM), and designated as L(Glu/Mal). Oxidative phosphorylation through complex I was subsequently stimulated by the addition of ADP (1 mM) and the resulting respiration state was recorded as P(C I). To achieve convergent electron input into the electron transport chain through complexes I and II, succinate (12 mM) was added, and the corresponding phosphorylating respiration state was recorded as P(C I+II). Outer mitochondrial membrane integrity was assessed by the addition of cytochrome c (32 µM). Respiration measured following cytochrome c supplementation was designated as P((C I+II)+Cyt c). The cytochrome c effect was calculated as the ratio of respiration between respiration rates after and before cytochrome c addition in the P(CI+II) state. An increased cytochrome c effect was interpreted as evidence of partial impairment of outer mitochondrial membrane integrity. To inhibit adenine nucleotide translocase activity, carboxyatractyloside (1 nM) was added, and the residual non-phosphorylating respiration was recorded as L(CAT). This state was used to estimate oxygen consumption unrelated to ATP synthesis and to evaluate mitochondrial coupling efficiency. The respiratory control index (RCI) values were calculated as the ratio of phosphorylating respiration P(C I) to leak respiration L(Glu/Mal).

Respiratory data acquisition and analysis were performed using DatLab software, version 7.4.0.4 (OROBOROS Instruments).

### 4.6. Statistical Analysis

Data are presented as mean ± SEM. Two-tailed paired Student’s *t*-tests were used for within-group comparisons, including changes from baseline in the same animals and comparisons within the same mitochondrial preparation. Two-tailed unpaired Student’s *t*-tests were used for between-group comparisons, including control vs. 2AF, control vs. 5MFF, and 2AF vs. 5MFF. For time-dependent haemodynamic parameters, comparisons were performed at individual time points relative to baseline or between treatment groups, as appropriate. Differences were considered statistically significant at *p* < 0.05.

## 5. Conclusions

This study provides the first in vivo evidence that 2AF and 5MFF exert acute hypotensive effects. Although both compounds produced a comparable reduction in arterial pressure, their divergent effects on heart rate and pulse pressure suggest partly different cardiovascular mechanisms. The mitochondrial findings further show that both compounds impair oxidative phosphorylation and affect mitochondrial membrane-associated functions in isolated rat liver mitochondria. Overall, 2AF and 5MFF can be identified as bioactive furan compounds with dual haemodynamic and bioenergetic actions.

## Figures and Tables

**Table 1:** Mean arterial pressure (mmHg, mean ± SEM) at different times after intraperitoneal administration of 5-methylfurfural (5MFF) or 2-acetylfuran (2AF).

Time After Injection, min	5MFF (*n* = 5)	2AF (*n* = 4)
0	96.6 ± 11.0	105.6 ± 10.5
0.5	85.8 ± 11.0	101.0 ± 11.4
1	84.5 ±10.9	93.0 ± 14.5
3	81.6 ± 11.2	77.3 ± 17.2 *
5	79.4 ± 10.1 *	70.3 ± 19.0 *
10	70.0 ± 8.0 *	60.6 ± 7.2 *
15	61.0 ± 9.2 *	64.3 ± 4.4 *
20	66.6 ± 6.1 *	68.1 ± 7.2 *
25	62.4 ± 6.9 *	73.4 ± 11.6 *
30	62.6 ± 6.5 *	75.3 ± 16.4 *

* *p* < 0.05 vs. pre-injection value (time 0).

## Data Availability

The original contributions presented in this study are included in the article. Further inquiries can be directed to the corresponding author.

## Supplementary Materials

The following supporting information can be downloaded at: https://www.mdpi.com/article/10.3390/ph19070995/s1, Supplementary Figure S1. Representative haemodynamic recordings obtained under control conditions (a) and after administration of 5-methylfurfural (5MFF) (b). SYS, systolic arterial pressure; DIAS, diastolic arterial pressure; MAP, mean arterial pressure. Arrow indicates the time of injection.

## Abbreviations

The following abbreviations are used in this manuscript:

5MFF	5-Methylfurfural
2AF	2-Acetylfuran
MAP	Mean arterial pressure
SYS	Systolic arterial blood pressure
DIAS	Diastolic arterial pressure
RCI	Respiratory control index
