# Supplementary material for: Acute Hypotensive Effects of 2-Acetylfuran and 5-Methylfurfural and Their Impact on Liver Mitochondrial Bioenergetics
Source: Pharmaceuticals (Basel). 2026 Jun 26;19(7):995. doi: 10.3390/ph19070995 (PMC13415649; doi:10.3390/ph19070995)
Supplement: Supplementary file 1 [file pharmaceuticals-19-00995-s001.zip › pharmaceuticals-4350468-supplementary.pdf]

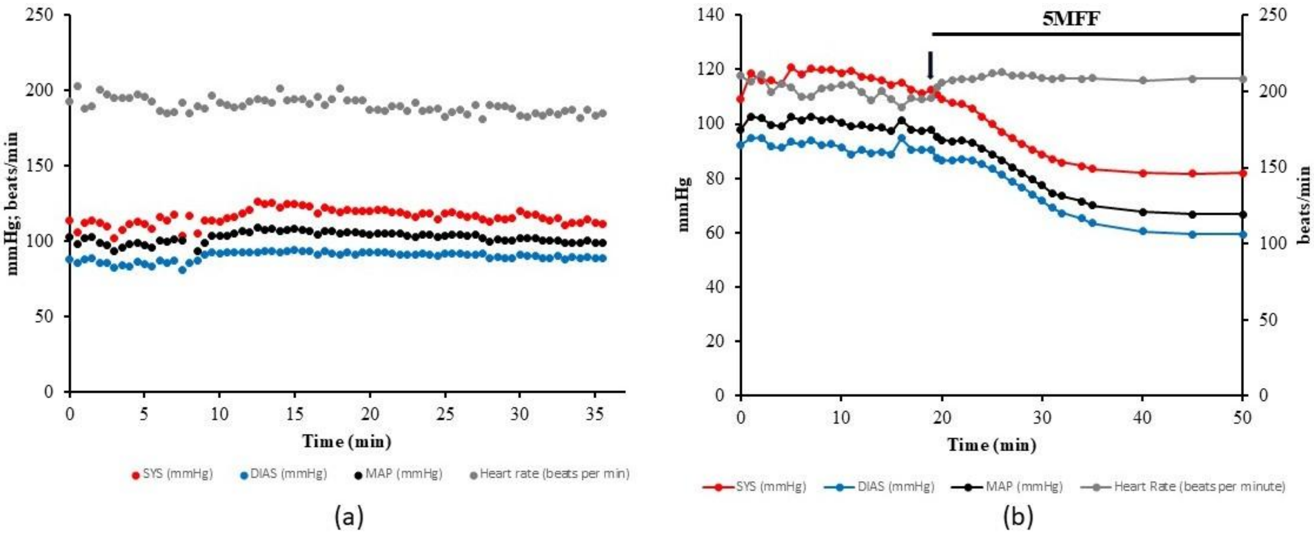

Supplementary Figure S1. Representative haemodynamic recordings obtained under control conditions (a) and after administration of 5-methylfurfural (5MFF) (b). SYS, systolic arterial pressure; DIAS, diastolic arterial pressure; MAP, mean arterial pressure. Arrow indicates the time of injection.
